# Some maternal lineages of domestic horses may have origins in East Asia revealed with further evidence of mitochondrial genomes and HVR-1 sequences

**DOI:** 10.7717/peerj.4896

**Published:** 2018-06-01

**Authors:** Hongying Ma, Yajiang Wu, Hai Xiang, Yunzhou Yang, Min Wang, Chunjiang Zhao, Changxin Wu

**Affiliations:** 1 College of Animal Science and Technology, China Agricultural University, Beijing, China; 2 Equine Center, China Agricultural University, Beijing, China; 3 Key Laboratory of Animal Genetics, Breeding and Reproduction, Ministry of Agriculture, Beijing, China; 4 State Key Laboratory for Conservation and Utilization of Bio-resources in Yunnan, Yunnan University, Kunming, China; 5 Institute of Genetics and Developmental Biology, Chinese Academy of Sciences, Beijing, China; 6 Institute of Animal Husbandry and Veterinary Science, Shanghai Academy of Agricultural Science, Shanghai, China; 7 Beijing Key Laboratory for Genetic Improvement of Livestock and Poultry, Beijing, China

**Keywords:** Horse mitochondrial genome, HVR-1, Divergence time, East Asia, Genetic origins

## Abstract

**Objectives:**

There are large populations of indigenous horse (*Equus caballus*) in China and some other parts of East Asia. However, their matrilineal genetic diversity and origin remained poorly understood. Using a combination of mitochondrial DNA (mtDNA) and hypervariable region (HVR-1) sequences, we aim to investigate the origin of matrilineal inheritance in these domestic horses.

**Methods:**

To investigate patterns of matrilineal inheritance in domestic horses, we conducted a phylogenetic study using 31 *de novo* mtDNA genomes together with 317 others from the GenBank. In terms of the updated phylogeny, a total of 5,180 horse mitochondrial HVR-1 sequences were analyzed.

**Results:**

Eightteen haplogroups (Aw-Rw) were uncovered from the analysis of the whole mitochondrial genomes. Most of which have a divergence time before the earliest domestication of wild horses (about 5,800 years ago) and during the Upper Paleolithic (35–10 KYA). The distribution of some haplogroups shows geographic patterns. The Lw haplogroup contained a significantly higher proportion of European horses than the horses from other regions, while haplogroups Jw, Rw, and some maternal lineages of Cw, have a higher frequency in the horses from East Asia. The 5,180 sequences of horse mitochondrial HVR-1 form nine major haplogroups (A-I). We revealed a corresponding relationship between the haplotypes of HVR-1 and those of whole mitochondrial DNA sequences. The data of the HVR-1 sequences also suggests that Jw, Rw, and some haplotypes of Cw may have originated in East Asia while Lw probably formed in Europe.

**Conclusions:**

Our study supports the hypothesis of the multiple origins of the maternal lineage of domestic horses and some maternal lineages of domestic horses may have originated from East Asia.

## Introduction

Domestication of horses has always attracted scientific interests as the animals played an important role in the civilization of humans in ancient times. Archaeological evidence indicated that the earliest domestication occurred in the Eurasian Steppe about 5,800 years ago (Outram & Stear, 2009). However, detailed information about the whole domestication process, including the subsequent domestication of horses, and the genetic origins of modern horses are still largely debated and need to be illustrated (Vilà & Leonard, 2001; Jansen & Forster, 2002; McGahern & Bower, 2006). Attributed to rapid molecular evolution and maternal inheritance, mitochondrial DNA (mtDNA) has been widely employed in phylogenetic studies of domestic horses, which revealed some valuable clues about the origins of modern horses (Lippold & Matzke, 2011; Achilli & Olivieri, 2012).

Due to the high number of polymorphic sites in the hypervariable regions of mtDNA, the first hypervariable region (HVR-1) was regarded as a useful tool for phylogenetic and phylogeographic studies. Vilà & Leonard (2001) constructed a neighbor-joining (NJ) tree using a total of 191 contemporary horses sampled across the world, in addition with eight ancient horse samples. The phylogenetic analyses uncovered at least six genetic clusters. Based on the results of Vilá et al., Jansen et al. studied the HVR-1 sequences of 318 horses from 25 breeds, and found 17 haplotype groups (Jansen & Forster, 2002). Subsequently, McGahern & Bower (2006) identified two new haplotypes in groups A and F, and expanded the number of HVR-1 haplotypes to 19. To obtain more accurate results of phylogenetic analyses, whole genomes of horse mitochondria have been used in the evolutionary studies of horses in recent years. In addition, Lippold & Matzke (2011) investigated the whole mitochondrial genomes of 59 domestic horses from 44 breeds and a Przewalskii horse (Equus przewalskii: a wild sub-species of horse). The result uncovered at least 46 mtDNA lineages in domestic horses, of which 73% already existed prior to horse domestication (Lippold & Matzke, 2011). Achilli & Olivieri (2012) analyzed 83 mitochondrial genomes of modern horses across Asia, Europe, and America. The results revealed 18 major haplogroups (A–R) and a more accurate haplotype divergence time. The aforementioned studies supported the hypothesis of multiple origins of domestic horse maternal lineages. However, some studies (e.g., Lei & Su, 2009; Yang & Zhu, 2017) proposed that certain horse lineages may have origins outside of the Eurasian Steppe. This suggests that subsequent domestication occurred after the first domestication event, which is supported by the fact that modern horses possess rich maternal lineages, referring to multiple origins of maternal lineages of the domesticated horses. If there was only one origin of domesticated horses, the diversity of mtDNA from the domesticated horses would be rather low. Contrary to the high diversity of mtDNA, extremely low diversity of DNA sequences of horse Y chromosome was detected in modern horses (Lindgren & Backström, 2004; Ling & Ma, 2010; Wallner & Piumi, 2004; Wallner & Vogl, 2013). Very limited horse paternal lineages can be attributed to extensive selection imposed on male horses after the domestication as a previous study of ancient DNA revealed abundant Y chromosome diversity in wild horses, which suggested that both male and female horse populations harbored rich genetic diversity before domestication (Lippold & Knapp, 2011).

Most previous phylogenetic studies on horses focused on horses from western countries. Although there are more than 5 million indigenous horses in China, the investigation of the origins of the local horses is still insufficient yet. Some studies indicated that the F haplogroup of horse HVR-1 may have originated in East Asia (Lei & Su, 2009; Cai & Tang, 2009). Recently, Yang et al. conducted a phylogenetic study on Chinese indigenous horses with HVR-1 sequences and found two new haplogroups, H and I. This study leads to increase in the number of the HVR-1 haplogroups of horse to 21, and yet, suggests East Asia origin for the two new haplogroups (Yang & Zhu, 2017). However, all of the above studies were confined to the HVR-1 of mtDNA.

The short mtDNA segment of HVR-1 is often accompanied by high levels of recurrent mutations, which blur the structure of the phylogenetic tree and render the distinction between some important branches within the tree virtually impossible (Achilli & Bonfiglio, 2009; Taberlet & Valentini, 2008; Torroni & Achilli, 2006). To address this issue as well as to improve the resolution of the matrilineal genealogical analyses, we combined the whole mtDNA genomes and HVR-1 sequences. Using this approach, we established the corresponding relationship between the haplogroups of mitochondrial genome and HVR-1 sequences and further analyzed the geographic distribution of the maternal lineages of the modern horses. Our analysis shows that East Asia was a genetic pool that may have provided the maternal lineages for domestic horses.

## Materials and Methods

### Samples and DNA extraction

Blood samples of 31 horses were collected from 14 Chinese domestic breeds across northern, northwestern and southwestern China (Table S1). The total genomic DNA was extracted using the phenol/chloroform extraction method. The total genomic DNA samples were further diluted to 50 ng/µl for the polymerase chain reaction (PCR) and DNA sequencing. All experimental procedures and protocols for sample collections were approved by the State Key Laboratory for Agro-biotechnology of CAU (Permit Number: XK257).

### PCR amplification and mitochondrial DNA sequences

To amplify the whole mitochondrial DNA sequences of the Chinese horses, primers were designed according to the published sequence X79547 (Xu & Arnason, 1994) (Table S2). All of PCR amplifications were conducted in a 20 µl volume containing 10 µl MasterMix (TIANGEN, Beijing, China), 8 µl double distilled water (ddH_2_0), 0.5 µl each primer (20 pmol/µl), and approximately 50 ng DNA. The PCRs were carried out as follows: 10 min of initial denaturation at 95 °C, followed by 30 cycles of 30 s at 95 °C, 60 s at annealing temperature (Table S2), and 30 s at 72 °C, with a final extension of 10 min at 72 °C. PCR products were directly sequenced using ABI 3730xl Genetic analyzer (Applied Biosystems, Foster City, CA, USA) by BGI-Tech Company (Beijing, China). In cases where samples were not amplified in the first attempt, re-amplification was performed. Sequences were deposited in the GenBank and accession numbers were listed in Table S1 .

We evaluated 31 *de novo* mitochondrial genomes with 317 others from the GenBank plus 16 sequences belonging to 16 Przewalskii horses (*Equus przewalskii*). The related information of the whole mtDNA sequences, including the geographical distribution and breed/type (where applicable), was provided in Table S3.

The HVR-1 sequences (5,180 sequences) in our study include: (1) 31 HVR-1 sequences isolated from the Chinese indigenous horses sequenced in this study (Table S1); (2) 317 HVR-1 sequences truncated from the whole mtDNA sequences obtained from Genbank (Table S4); and (3) 4,832 sequences retrieved from GenBank (Table S5).

### Sequencing data analysis

By using MitoToolPy (http://www.mitotool.org/mp.html) (Peng & Fan, 2015), we scored the variants for each sequence (whole mtDNA genome sequences) relative to the reference sequence JN398377 (Achilli & Olivieri, 2012). We assigned the mtDNA sequences into specific haplogroups according to DomeTree (http://www.dometree.org/trees/horse.htm) (Peng & Fan, 2015). We followed the caveats for quality control in mtDNA dataset of domestic animals (Shi & Fan, 2014).

### Phylogenetic reconstruction

To depict the phylogeographic patterns for whole mtDNA sequence, we assigned each of the 348 whole mtDNA sequences into haplogroups in context of updated haplogroup tree according to DomeTree (http://www.dometree.org/trees/horse.htm) (Peng & Fan, 2015). This tree encompasses 348 sequences and was rooted with donkey (*Equus asinus*) sequence.

NETWORK 5.0 (http://www.fluxus-engineering.com) was used for calculation of the median joining network for HVR-1 sequences. Calculations were performed under the following conditions: (1) default setting of Epsilon (0) was chosen; (2) three mutational hotspot sites 15585,15597 and 15650 were set as zero, and two mutational hotspot sites 15659,15737 were downweighted (weight :5) (Jansen & Forster, 2002; Cieslak & Pruvost, 2010); (3) the “>1 frequency” setting in MJ calculation was activated and MP Calculation option was conducted to remove excessive links and median vectors; and (4) other parameter settings were maintained as defaults.

Haplotype partitioning was quantified by DnaSP v5.0 software (Librado & Rozas, 2009). To verify the results of Network, we also assigned each of the haplotypes of HVR-1 sequences into haplogroups in context of updated mitogenome haplogroup tree according to DomeTree (http://www.dometree.org/trees/horse.htm) (Peng & Fan, 2015).

### Molecular dating

Using 348 mtDNA genomes, we inferred the time of the most recent common ancestor of the haplogroups as employed in BEAST v1.8 (Drummond & Rambaut, 2007; Drummond & Suchard, 2012). We used GTR+I+G substitution model selected by jModelTest 2.1.4 (Darriba & Taboada, 2012; Guindon & Gascuel, 2003). The mutation rate of 7.39 × 10^−2^ mutations/site/Mya (95% confidence interval 2.49 × 10^−2^–1.60 × 10^−1^) (Lippold & Matzke, 2011) was used under both strict and relaxed clock models (Drummond & Rambaut, 2005). Each Markov chain Monte Carlo was run for 60,000,000 generations and the first 10% of each run was discarded as burn-in, and sampled every 1,000 generations. We confirmed convergence with Effective Sample Size values >300 in Tracer v1.6 (Drummond & Rambaut, 2007). We then used TreeAnnotator v1.6.1 implemented in the BEAST for the phylogenetic tree summaries and viewed the final tree with attributes such as posterior, node age, and height median in FigTree v1.4.3 (http://tree.bio.ed.ac.uk/software/figtree/).

## Results

### Haplogroup tree and network

**Figure 1:**
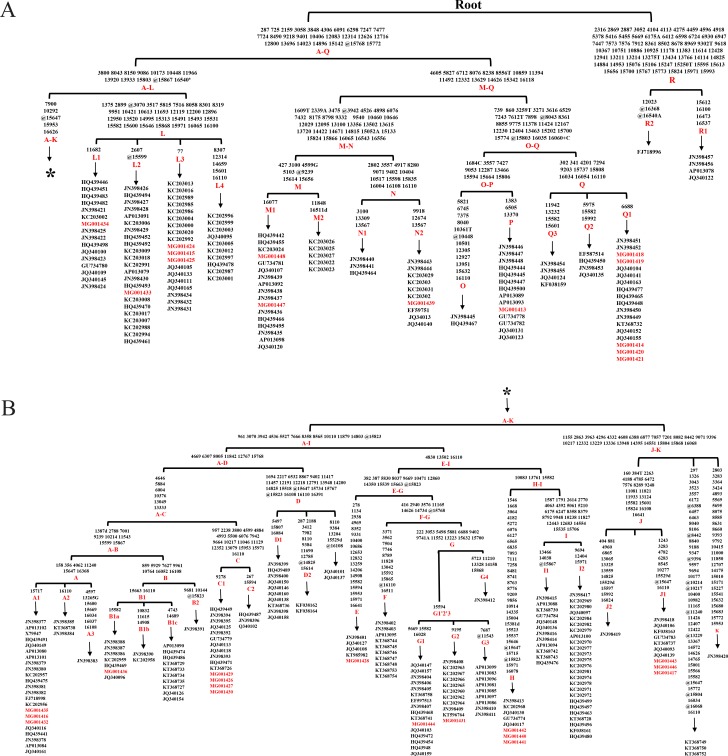
Haplogroup tree of mtDNA genome sequences from the studied horses. Mutations were shown on the branches and were numbered according to a horse reference sequence (JN398377). Mutations toward a base identical-by-state to the JN398377 were indicated with the prefix. “W”, “S”, “M”, “K”, “R” and “Y” specifying the heteroplasmic status of A/T, C/G, A/C, G/T, A/G and C/T at a certain site, respectively. Deletions were indicated by a “d” after the deleted nucleotide position. Insertions are indicated by a “+” followed by the position number and type of inserted nucleotide(s). Repetitive align region np 16126-16352 are not considered. The information of horses in each clade was provided in Table S3 (The red Genebank numbers indicate 31 *de novo* mtDNA genome sequence); (B) is a continuation of (A) from the position of the asterisk.

The analysis of whole mtDNA sequences revealed 16 clades (Aw-Rw) according to DomeTree (http://www.dometree.org/trees/horse.htm) (Peng & Fan, 2015) (Fig. 1). The system of nomenclature of those haplogroups was in accordance with Achilli & Olivieri (2012). The result showed that horses from the same breeds and/or regions were widely distributed within the clades. Further, the resulting matrilineal genealogy does not show evidence of strong concordance with breeds and/or regions. This therefore may support the hypothesis that horse may have multiple origins of matrilineal inheritance.

**Figure 2:**
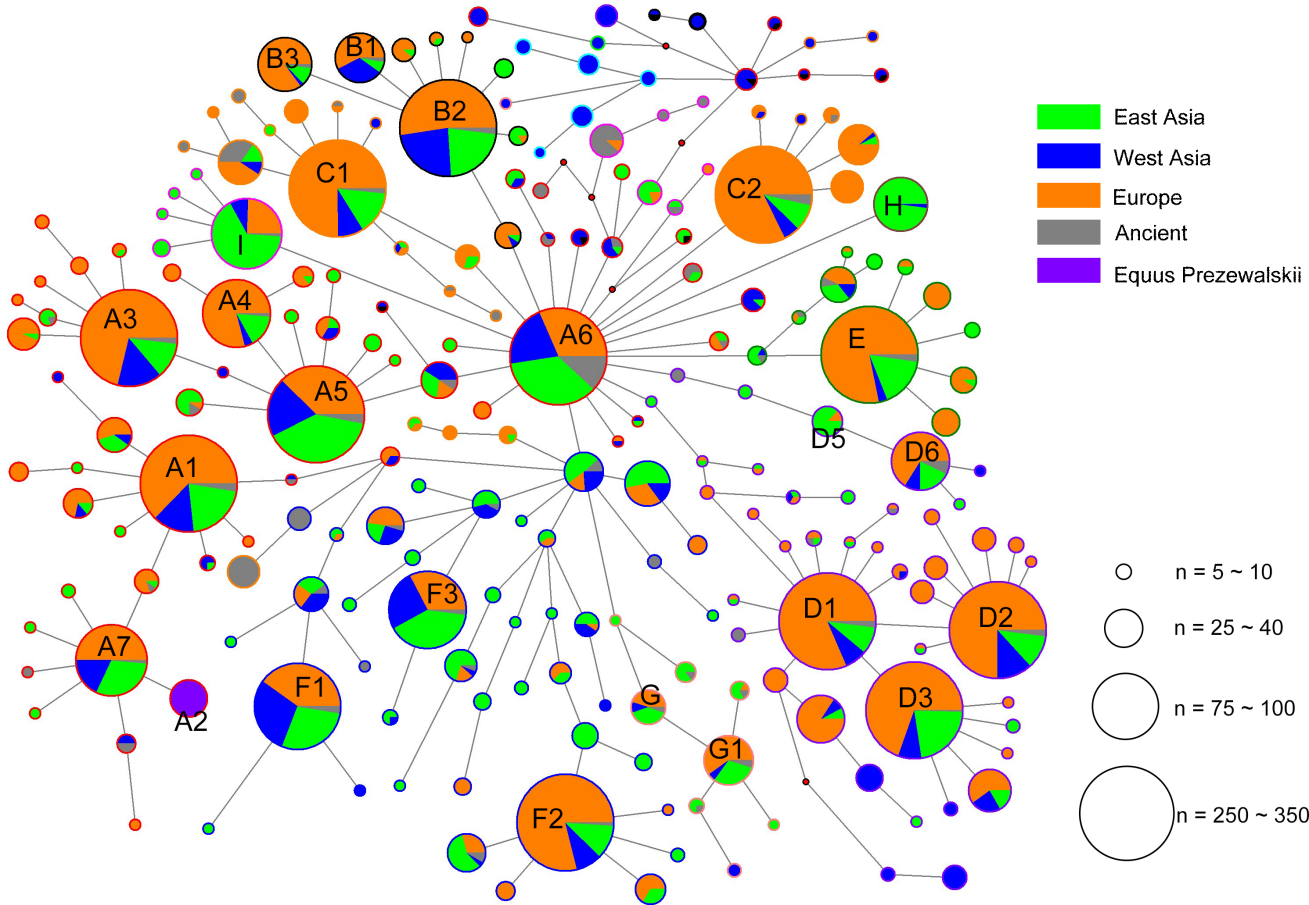
Median joining network constructed based on the 247- bp HVR-1 sequences. Circles are proportional to the number of horses represented and a scale indicator (for node sizes) was provided. The length of lines represents the number of variants that separate nodes (some manual adjustment was made for visually good). In the circles, the colors of solid pie slices indicate studied horse populations: Orange, European horses; Blue, horses of West Asia; Light Green, horses from East Asia; Grey, ancient horses; Purper, Przewalskii horses.

The alignment of the 5,180 HVR-1 sequences produced 397 haplotypes. We identified three strong mutational hotspots which have been previously described at nucleotide positions 15585, 15597, and 15650 (Vilà & Leonard, 2001; Jansen & Forster, 2002). These positions were not included in our phylogenetic analyses. In addition, two mutational hotspots 15659, 15737 were individually down-weighted (weight 5). Once these mutational hotspots were eliminated from the dataset, we divided the sequences into nine haplogroups from A to I based on the structure of the network (Fig. 2), of which haplogroups H and I were identified in our previous study (Yang & Zhu, 2017), and the four clusters B3, D5, D6, and G1 were newly observed in the present study.

**Figure 3:**
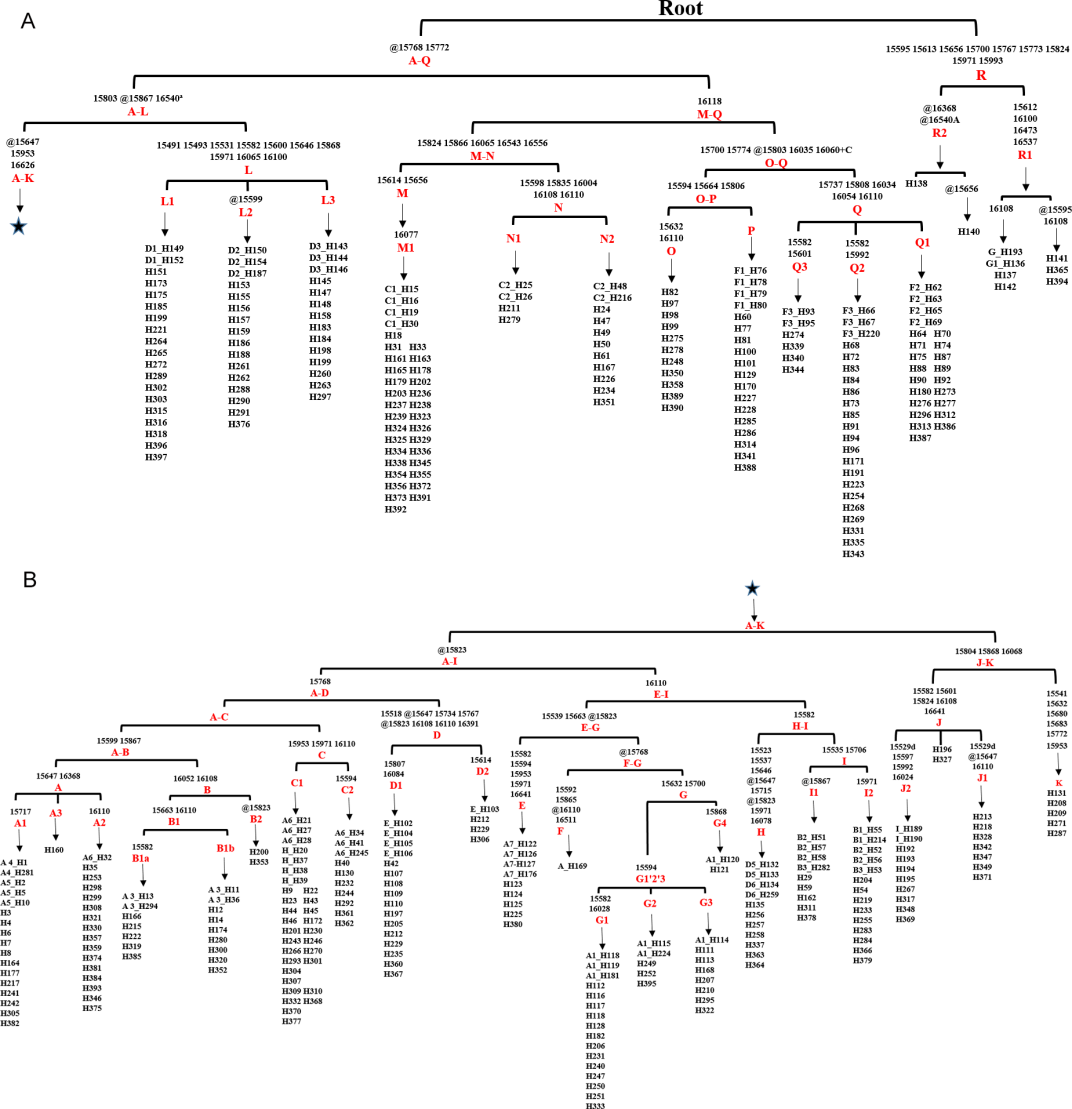
Haplogroup tree of HVR-1 sequences from the studied horses. Mutations were shown on the branches and were numbered according to a horse reference sequence (JN398377). Repetitive align region np 16126–16352 are not considered. The information of horses in each clade was provided in Table S5. This haplogroup tree showed the corresponding relationship between the haplogroups of the horse mitogenomes and HVR-1 sequences from the studied horses; (B) is a continuation of (A) from the position of the asterisk.

Based on the result of Network we found a small proportion of HVR-1 haplogroups, which were out of the scope of A-I haplogroups. For completeness those we assigned each of the haplotypes of HVR-1 sequences into haplogroups in context of updated mitogenome haplogroup tree according to DomeTree (http://www.dometree.org/trees/horse.htm) (Peng & Fan, 2015), results were showed in Fig. 3 and Table S5.

### Corresponding relationships between haplogroups of mtDNA HVR-1 and whole genomes

To establish the relationships between the haplotypes defined from HVR-1 and whole mtDNA sequences, we retrieved the HVR-1 sequences from the 348 whole mitochondrial genomes and conducted phylogenetic analysis (Table S4). A clear corresponding relationship was revealed when comparing the haplotypes of HVR-1 and mitochondrial genomes of all the samples in Table 1(except three Przewalskii horse and one ancient Yakut horse). A haplogroup of the genome corresponded to one or more of its HVR-1 sequence counterparts. The haplogroup A is the clade containing the largest number of haplotypes defined on the basis of HVR-1, but its sub-haplogroups clustered into 6 haplogroups of mitochondrial genomes. L_w_ contained three sub-haplogroups of HVR-1 and is the largest haplogroup containing most haplotypes of the mitochondrial genome.

**Table 1:** Corresponding relationship between the haplogroups defined with the mitochondrial genome and the HVR-1 sequences. A haplogroup of the genome corresponded to one or more of its HVR-1 sequence counterparts. The haplogroup A is the clade containing the largest number of haplotypes defined on the basis of HVR-1, but its sub-haplogroups are clustered into six haplogroups of mitochondrial genomes. L_w_ contained three sub-haplogroups of HVR-1 and is the largest haplogroup containing most haplotypes of the mitochondrial genome.

Hg_W_*	A_w_	B_w_	C_w_	D_w_	E_w_	F_w_	G_w_	H_w_
Hg_H_*	A4,A5,A6(less)	A3	H,A6(most)	E	A7	A2	A1	D5,D6
Hg_W_*	I_w_	J_w_ K_w_	L_w_	M_w_	N_w_	O_w_P_w_Q_w_		R_w_
Hg_H_*	B1,B2,B3	I	D1,D2,D3	C1	C2	F1-F3		G1

**Notes.**

*Hg_W_ haplogroup of whole mtDNA sequence *Hg_H_ haplogroup of HVR-1 sequence

### Geographic distributions of horse mtDNA haplogroups

The analysis of geographic distribution of the mitochondrial genome haplogroups showed that horse populations in Europe or East Asia included all haplogroups defined from the mtDNA genome sequences. The lineage F_w_ comprised entirely of Przewalskii horses. The two haplogroups I_w_ and L_w_ displayed frequency peaks in Europe (14.08% and 37.32%, respectively) and a decline to the east (9.33% and 8.00% in the West Asia, and 6.45% and 12.90% in East Asia, respectively), especially for Lw, which contained the largest number of European horses (Table 2). However, an opposite distribution pattern was observed for haplogroups Aw, Hw, Jw, and Rw, which were harbored by more horses from East Asia than those from other regions. The proportions of horses from East Asia for the four haplogroups were 38%, 88%, 62%, and 54%, respectively.

**Table 2:** Geographic distribution of haplogroups of the mitochondrial genomes(%). The two haplogroups I_w_ and L_w_ displayed frequency peaks in Europe and a decline to the east especially for Lw, which contained the largest number of European horses. However, an opposite distribution pattern was observed for haplogroups Aw, Hw, Jw, and Rw, which were harbored by more horses from East Asia than those from other regions.

Clade of mtDNA genome	East Asia	West Asia^a^	Europe
A_w_	9.68	9.33	6.34
B_w_	4.03	9.33	5.63
C_w_	6.45	6.67	2.11
D_w_	7.26	1.33	2.82
E_w_	2.42	1.33	0.70
G_w_	12.90	12.00	9.86
H_w_	5.64	0	0.70
I_w_	6.45	9.33	14.08
J_w_	5.64	2.67	0.70
K_w_	0	0	0.70
L_w_	12.90	8.00	37.32
M_w_	5.64	4.00	8.45
N_w_	2.42	1.33	5.63
O_w_	2.42	6.67	0
P_w_	4.03	2.67	1.41
Q_w_	9.68	10.67	2.82
R_w_	2.42	1.33	0.70

**Notes.**

a It indicates the areas of West Asia and its adjacent regions in the present study.

Considering the large amount of HVR-1 sequence data of horse mtDNA currently available in the literatures or deposited in GenBank, we also analyzed the geographic distributions of the HVR-1 haplogroups (Table 3). We further confirmed our geographic distributions of the whole mtDNA haplogroups by analyzing the known mitochondrial HVR-1 sequences, using the corresponding relationships between the two sets of haplogroups (Table 1). The results showed that haplogroups D1, D2, and D3, which correspond to haplotype L_w_ of the whole mitochondrial sequences, displayed the highest frequencies in European horses, while I(corresponding to J_w_), G1 (corresponding to R_w_) showed frequency peaks in East Asia and a decline from Asia to Europe (Table 3). However, the patterns of geographic distribution of Aw and Hw were not supported by HVR-1 data. It should be noted that Cw contains H and majority of haplotypes in the HVR-1 haplogroup A6, which had a frequency peak in the West Asian regions. It is in accordance with geographic distribution of haplogroup A6, which is the most ancient haplogroup defined by HVR-1 sequences and contains the highest proportion of ancient horse sequences. However, haplogroup H has the highest frequency in the horse populations of East Asia.

**Table 3:** Geographic distribution of haplogroups of the HVR-1 sequences. The haplogroups D1, D2, and D3, which displayed the highest frequencies in European horses, while I, G1 showed frequency peaks in East Asia and a decline from Asia to Europe.

HVR-1 haplotype	East Asia	West Asia^a^	Europe	Ancient
A1	7.62	9.44	8.62	5.04
A2	0.93	1.55	0.03	0
A3	3.81	8.73	8.52	3.36
A4	1.10	0.56	2.33	0.42
A5	7.28	5.21	2.16	2.94
A6	7.96	11.97	2.26	15.55
A7	2.79	2.96	1.38	2.52
B1	0.85	2.11	1.05	0.42
B2	3.73	6.62	3.77	2.10
B3	1.02	0.85	1.54	0.42
C1	4.91	5.49	9.11	18.91
C2	2.88	2.68	9.44	4.62
D1	3.64	6.20	11.41	5.46
D2	3.90	6.34	10.82	3.36
D3	5.84	3.80	7.44	0.42
D5	1.35	0	0.07	1.68
D6	1.10	1.13	1.21	1.68
E	4.49	1.41	6.06	3.36
F1	6.86	10.56	2.43	3.78
F2	6.69	5.63	6.78	2.94
F3	7.37	4.08	1.41	8.82
G	1.10	0.70	0.29	0.42
G1	1.95	0.56	0.85	1.68
H	4.49	0.28	0.03	0
I	6.35	1.13	0.98	10.08

**Notes.**

a It indicates the areas of West Asia and its adjacent regions in the present study.

### Divergence times of haplogroups C_w_, J_w_, and R_w_

**Figure 4:**
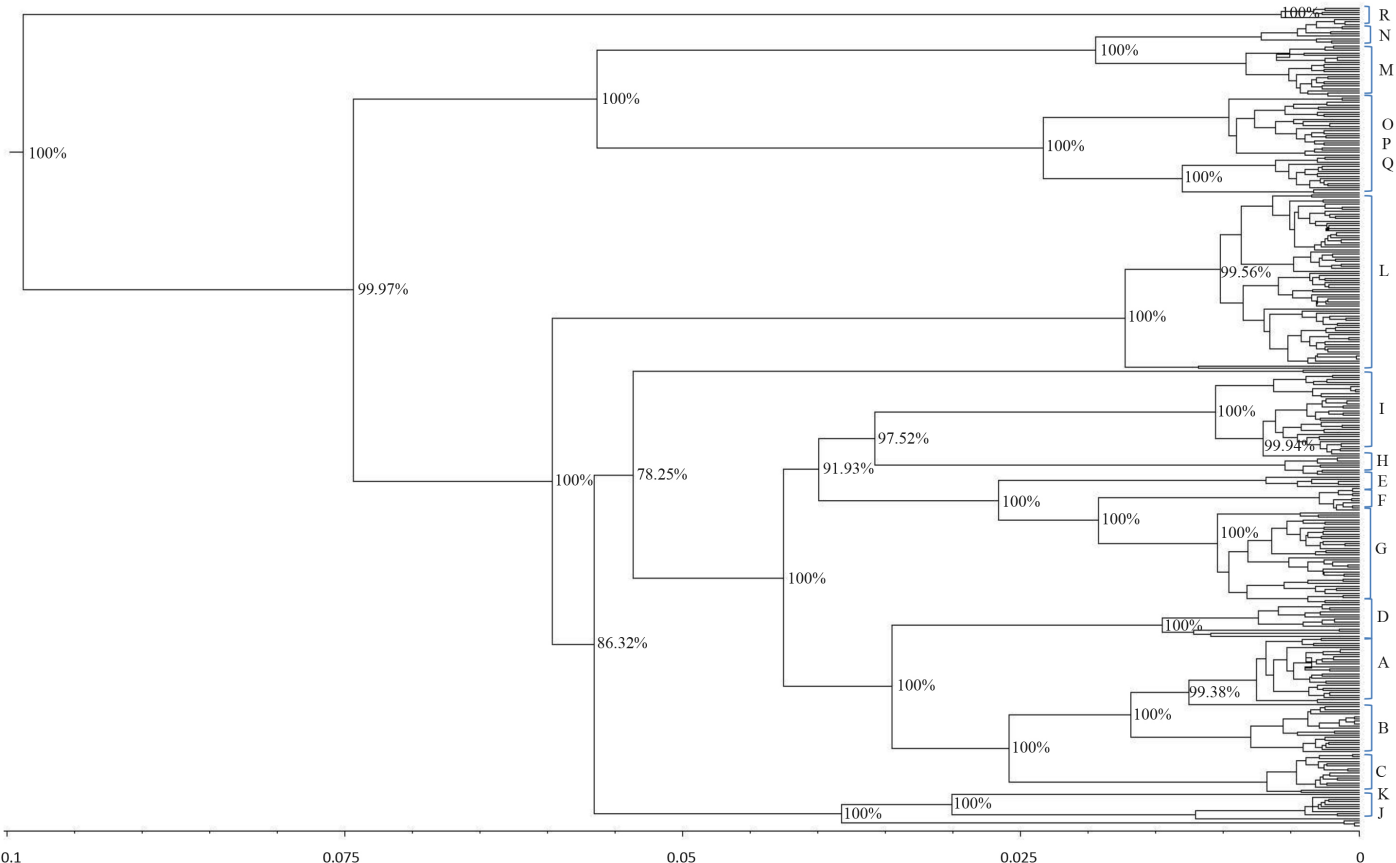
Schematic phylogeny of mtDNAs genome from modern horses. This tree includes 348 sequences and was rooted at a donkey (*E. asinus*) mitochondrial genome (not displayed). The topology was inferred by a beast approach, whereas a time divergence scale (based on rate substitutions) is shown on the bottom (age estimates were indicated with thousand years (KY)). The percentages on each branch represent Bayesian posterior credibility and the alphabets on the right represent the names of haplogroups. Additional details concerning ages were given in Tables S3 and S6.

The results showed that modern horses shared a common ancestor around 99,800 years ago. The phylogenetic tree for the estimation of the divergence time had 95% credibility intervals for the main branches. The resulting tree topology (Fig. 4) was very similar to the results obtained with the haplogroup tree of mtDNA genome sequences. Almost half of the haplotypes formed before the widely regarded time of the earliest horse domestication (approximately 5,800 years ago) (Outram & Stear, 2009).

From the phylogenetic analysis of both mitochondrial HVR-1 and genome sequences, haplogroups J_w_, R_w_, and some maternal lineage of Cw (H haplogroup defined by HVR-1) showed high frequency in East Asia. To further investigate their possible origins, we determined their divergence time. The age of haplogroup C_w_ was estimated to be 7.8 thousand years (KYA; 95% highest posterior density (HPD): 1,000–18,800 years), while the age of haplogroup J_w_ was 13.1 thousand years (KYA; 95% highest posterior density (HPD): 1,700–32,600 years) and haplogroup R_w_ has an age more than 6.7 thousand years (KYA; 95% highest posterior density (HPD): 900–16,600 years)(Table S6).

## Discussion

Polymorphism in mtDNA sequences have been widely used in the study of evolution due to its highly variable sites, multiple copy numbers, as well as ease of obtaining mtDNA from various samples. Mitochondrial HVR-1 sequences have been used in most studies on horse phylogenetics in the past, which revealed a high level of diversity harbored by maternal lineages of domestic horses that were contrary to the extremely low polymorphisms detected in the horse Y chromosome (Ling & Ma, 2010; Lindgren & Backström, 2004). This suggested that there were multi-origins of horse maternal lineages (Vilà & Leonard, 2001; Cai & Tang, 2009). These studies also showed the geographic patterns of some haplotypes of the HVR-1 sequences, which indicated that the events of horse domestication or the introgression of wild horses occurred in several places across the Asia-Europe continents (Lei & Su, 2009; Yang & Zhu, 2017).

However, the short sequence of the HVR-1 and relatively limited number of phylogenetically informative sites lead to low statistical support for most nodes of the phylogenetic tree of the domestic horses, which remains unresolved. To overcome this, whole mtDNA sequences were used in horse maternal evolutionary studies in recent years. This has resulted in high phylogenetic resolution and estimation of precise dates of divergence (Lippold & Matzke, 2011; Achilli & Olivieri, 2012). Our phylogenetic analysis of horse mitochondrial genomes revealed 18 haplogroups. We also named the haplogroups according to the study of Achilli & Olivieri (2012).

Studies (e.g., Lei & Su, 2009; Ding & Oskarsson, 2011) have indicated that the frequencies of certain haplotypes demonstrated a gradient in the geographical distribution and that areas with the highest frequencies may be related to the original domestication of the lineages. Previous analysis of the geographic distribution of the mitochondrial genome haplogroups showed that L_w_ had a frequency peak in Europe, suggesting that this haplogroup may have origins in Europe (Lippold & Matzke, 2011; Achilli & Olivieri, 2012). Our results also indicated that L_w_ was the most frequent haplogroup in European horses, which supported the previous hypothesis of the European origins of L_w_. Furthermore, we found that haplogroups J_w_ and R_w_ had frequency summits in the horses from East Asia. In our study, the three HVR-1 haplogroups D1, D2, and D3, corresponding to L_w_, which were closely clustered together (Fig. 2), showed a decline of frequency from Europe to Asia, which were in accordance with the results from previous studies on mitochondrial genome and HVR-1 sequences (Achilli & Olivieri, 2012; Lei & Su, 2009; Cai & Tang, 2009), while the HVR-1 haplogroup I (corresponding to J_w_), and G1 (corresponding to R_w_), and H (represented by C_w_), all showed a frequency peak in East Asia and declining frequencies to the west. Most horses that harbored the HVR-1 haplotypes H and I, which were represented by Cw and Jw, respectively, were indigenous horses of the South of China, which agrees with the results of our previous study (Yang & Zhu, 2017).

The analysis of divergence time showed that more than half of the maternal lineages formed before horse domestication, which supported the previous hypothesis of multi-origins of modern horses. Many haplotypes in C_w_, J_w_, and R_w_ also had divergence times before the domestication of wild horses (about 5,800 years ago), and most of those lineages belong to East Asia, especially distributing in south of China, the mountainous areas in the south of Yangtze River (Table S3), which may have served as places of refuge for wild horses during the Last Glacial Maximum (25–19.5 KYA) and the Younger Dryas (12.7–11.5 KYA) (Clutton-brock, 1990; Macfadden, 1995). These horses may have been domesticated later and contributed to the genetic pools of domestic horse maternal lineages.

## Conclusions

Our study supports the hypothesis that the multiple origins of the maternal lineages of domestic horses and some maternal lineages of domestic horses may have originated from East Asia.

##  Supplemental Information

10.7717/peerj.4896/supp-1 Table S1 The amplified sample information of the Chinese indigenous horses

10.7717/peerj.4896/supp-2 Table S2 Oligonucleotide primers for amplifying fragments of the horse mtDNA genomes

10.7717/peerj.4896/supp-3 Table S3 The sample information of the whole mtDNA sequences

10.7717/peerj.4896/supp-4 Table S4 The samples used for establishing the corresponding relationship between the haplogroups of the mitochondrial genome and HVR-1 sequences

10.7717/peerj.4896/supp-5 Table S5 The sample information of the horse mitochondrial HVR-1 sequences

10.7717/peerj.4896/supp-6 Table S6 Estimated coalescence ages of horse whole mtDNA haplogroups

## Additional Information and Declarations

### DNA Deposition

The following information was supplied regarding the deposition of DNA sequences: Sequences were deposited at GenBank Bank and can be located via the following accession numbers:
MG001413, MG001414, MG001415, MG001416, MG001417, MG001418, MG001419, MG001420, MG001421, MG001424, MG001425, MG001426, MG001427, MG001428, MG001429, MG001430, MG001431, MG001432, MG001433, MG001434, MG001435, MG001436, MG001439, MG001440, MG001441, MG001442, MG001443, MG001444, MG001446, MG001447, MG001448.

### Data Availability

The following information was supplied regarding data availability: The raw data are provided in the Supplemental Files.
